# Inverse Association Between Composite Dietary Antioxidant Index and Prevalence of Pelvic Inflammatory Disease Among Women: A Cross-Sectional Study of NHANES 2013–2018

**DOI:** 10.3390/healthcare14121682

**Published:** 2026-06-12

**Authors:** Yuhang Liu, Gu Hu, Ziyue Zhou, Shuaibin Liu

**Affiliations:** 1 The Second Clinical College, Chongqing Medical University, Chongqing 400010, China; 2024210102@stu.cqmu.edu.cn (Y.L.); 2024210020@stu.cqmu.edu.cn (G.H.); 2 Xinglin College, Liaoning University of Traditional Chinese Medicine, Shenyang 110032, China; 18848212108@163.com; 3 The Second Affiliated Hospital of Chongqing Medical University, Chongqing 400010, China

**Keywords:** composite dietary antioxidant index (CDAI), pelvic inflammatory disease (PID), National Health and Nutrition Examination Survey (NHANES), cross-sectional study, machine learning

## Abstract

**Background:** Pelvic inflammatory disease (PID) is a prevalent chronic inflammatory condition among women. The Composite Dietary Antioxidant Index (CDAI), a measure of dietary antioxidant capacity, has been associated with various inflammatory diseases, but evidence concerning its association with PID remains limited. **Methods:** The final analytic sample included 4539 women. CDAI was calculated from six dietary antioxidant components: vitamin A, vitamin C, vitamin E, carotenoids, zinc, and selenium. Survey-weighted multivariable logistic regression models were used to evaluate the association between CDAI and self-reported history of treated PID, incorporating the sampling weights, strata, and primary sampling units of NHANES. Restricted cubic spline (RCS) analysis was used to assess both linear and non-linear associations. Subgroup analyses and a machine learning model based on random forest, combined with SHapley Additive exPlanations (SHAP) value ranking, were conducted to evaluate the relative importance of individual components of CDAI. **Results:** In the fully adjusted spline model including smoking status, CDAI was inversely associated with the odds of self-reported history of treated PID, with no statistical evidence of nonlinearity. Compared with the lowest quartile (Q1), the odds ratios (ORs) for self-reported history of treated PID across higher quartiles of CDAI were as follows: Q2 (OR = 0.682, 95% CI: 0.485–0.959, *p* = 0.036), Q3 (OR = 0.524, 95% CI: 0.334–0.819, *p* = 0.009), and Q4 (OR = 0.666, 95% CI: 0.380–1.167, *p* = 0.167). Among the components of CDAI, vitamin E intake showed an independent inverse association with the odds of self-reported history of treated PID. The SHAP value interpretation indicated that vitamin A, vitamin C, and carotenoids were the three components in CDAI with the highest predictive contribution. Furthermore, subgroup analysis demonstrated a significant interaction effect of age on the association between CDAI and PID. **Conclusions:** This cross-sectional study suggests an inverse association between CDAI and self-reported history of treated PID, particularly in spline analyses; however, the quartile-based fully adjusted results were non-monotonic and attenuated after adjustment for smoking status. These findings provide hypothesis-generating evidence for future longitudinal and mechanistic studies on antioxidant-related dietary patterns and PID-related reproductive health.

## 1. Introduction

PID is a common inflammatory condition primarily caused by Chlamydia trachomatis, Neisseria gonorrhoeae, and pathogens associated with bacterial vaginosis [1,2]. Its clinical manifestations include persistent lower abdominal pain, abnormal vaginal discharge, and other symptoms. PID predominantly affects sexually active young women, with an average age of onset around 25 years. If not diagnosed and treated promptly, PID can lead to ectopic pregnancy, infertility, chronic pelvic pain, and other adverse pregnancy outcomes [3,4,5], significantly impacting women’s quality of life. Therefore, early identification and intervention are crucial. Recent international studies and guidelines further indicate that PID is not only related to classical sexually transmitted pathogens such as Chlamydia trachomatis and Neisseria gonorrhoeae, but is also frequently polymicrobial and may involve Mycoplasma genitalium, bacterial vaginosis-associated anaerobes, and vaginal microbiota disruption. These findings highlight the complexity of PID etiology and the need to consider host-related and modifiable factors, including diet-related inflammatory and antioxidant exposures [1,2,5,6].

In recent years, antioxidant diets have garnered widespread attention. CDAI is a quantitative measure that comprehensively evaluates the antioxidant capacity of the diet, encompassing six nutrients: vitamins A, C, E, carotenoids, zinc, and selenium [7]. Previous studies have shown that CDAI is positively associated with various health indicators such as hypertension, diabetes, coronary heart disease, non-alcoholic fatty liver disease, sleep quality, and grip strength [8,9,10,11,12,13]. Although observational studies suggest that CDAI may be associated with inflammation-related outcomes [14,15], evidence regarding its association with PID remains limited. Evidence directly examining antioxidant-related dietary exposures and PID is emerging but remains limited. A recent nationwide survey and Mendelian randomization study among U.S. women reported that higher CDAI was associated with lower PID prevalence, although its Mendelian randomization analysis did not support a clear causal effect of individual antioxidants [16]. In addition, NHANES-based studies have reported inverse associations between dietary copper intake and PID prevalence, as well as between dietary magnesium intake and PID prevalence [17,18]. These findings suggest that antioxidant-related dietary factors may be relevant to PID susceptibility; however, most available evidence is observational, and the relative contribution of individual CDAI components remains insufficiently characterized. Mechanistically, antioxidant intake may influence PID-related inflammatory susceptibility and tissue response by modulating oxidative stress, epithelial and mucosal barrier integrity, and immune-inflammatory signaling. Vitamins A, C, and E, carotenoids, selenium, and zinc can reduce reactive oxygen species, limit lipid peroxidation, and regulate redox-sensitive pathways such as NF-κB and MAPK, thereby potentially suppressing inflammatory mediators including TNF-α, IL-6, iNOS, COX-2, and prostaglandin E2 in pelvic tissues [19]. Selenium and zinc are also involved in antioxidant enzyme activity and immune-cell regulation, whereas vitamin C may interact with vitamin E within the antioxidant recycling network [20,21,22,23,24,25]. These pathways provide biological plausibility for an inverse association between CDAI and PID prevalence.

Based on this, this study utilizes cross-sectional data from the NHANES 2013–2018 to systematically evaluate the association between CDAI and PID. Furthermore, machine learning methods (random forest) are employed to explore the relative importance of CDAI components in predicting PID. This study aims to provide population-based observational evidence to inform future prospective and mechanistic research on diet-related antioxidant exposure and PID.

## 2. Methods

### 2.1. Study Population

The National Health and Nutrition Examination Survey (NHANES) is a nationally representative health monitoring program conducted by the National Center for Health Statistics (NCHS), which is part of the Centers for Disease Control and Prevention (CDC), aiming to comprehensively assess the health and nutritional status of the U.S. population. A total of 29,400 participants from NHANES 2013–2018 were initially considered. We first restricted the analytic sample to women who were eligible for the reproductive health questionnaire item RHQ078 according to the NHANES design. Among these eligible participants, women with missing, refused, or “don’t know” responses to RHQ078, as well as those with missing data required for CDAI calculation or smoking status, were further excluded. The final analytic sample comprised 4539 women.

The NHANES protocols were approved by the NCHS Research Ethics Review Board, and all participants provided written informed consent. The present analysis used de-identified public-use data and required no additional institutional review board approval. In addition, written informed consent was provided by all participants, in accordance with ethical standards.

**Figure 1:**
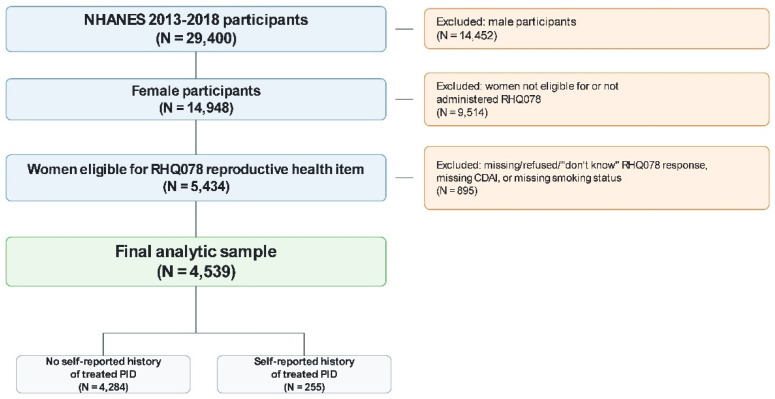
Flowchart of participant selection from NHANES 2013–2018.

Figure 1 presents the flowchart of participant selection, including the restriction to the RHQ078-eligible target population and subsequent exclusion of eligible participants with incomplete outcome or covariate data.

### 2.2. Measurement of CDAI

In this study, the CDAI was used as the primary exposure variable to assess an individual’s dietary antioxidant capacity. The CDAI was constructed based on data from the 24 h dietary recall surveys in the NHANES database, a tool widely used in nutritional epidemiological research. In the current study, all participants completed two 24 h dietary recalls: the first was conducted through face-to-face interviews by trained interviewers, and the second was completed via telephone 3 to 10 days later. For each antioxidant component, the mean intake across the two available non-consecutive 24 h dietary recalls was used before standardization and CDAI calculation. No formal usual-intake modeling framework, such as the National Cancer Institute method or the Multiple Source Method, was applied. Therefore, the CDAI in this study should be interpreted as an average of reported short-term dietary intake rather than a modeled estimate of long-term habitual antioxidant intake. Because short-term dietary recalls are affected by within-person day-to-day variation and recall error, some degree of non-differential exposure misclassification is possible, which may have attenuated the observed associations. Respondents were required to recall in detail all food and beverages consumed during the previous 24 h. The survey data were transmitted electronically to the SurveyNet system, and nutrient intakes were calculated using the USDA Food and Nutrient Database for Dietary Studies (FNDDS) [26,27].

The CDAI was calculated based on six antioxidant components: vitamin A, vitamin C, vitamin E, zinc, selenium, and carotenoids. To compute the CDAI, the methodology proposed by Wright et al. was followed [28]. The intake levels of the aforementioned six antioxidants were standardized by subtracting the mean and dividing by the standard deviation, and the standardized values for each nutrient were summed to obtain the CDAI [29]. The specific formula for calculating CDAI is as follows:

$$CDAI=\sum_{i=1}^{6}\left(\frac{IndividualIntake-Mean}{SD}\right)$$

### 2.3. Assessment of the Diagnosis of PID

PID status was defined based on the NHANES Reproductive Health Questionnaire item RHQ078, which asked: “Have you ever been treated for an infection in your fallopian tubes, uterus, or ovaries, also called a pelvic infection, pelvic inflammatory disease, or PID?” Participants who answered “Yes” were classified as having a self-reported history of treated PID.

### 2.4. Covariates

Based on previous research and potential confounding factors, we selected the following variables as covariates in our analysis [17]: demographic variables (age, race/ethnicity, education level, marital status); socioeconomic status [Poverty Income Ratio (PIR)]; health-related lifestyle indicators [Body Mass Index (BMI), smoking status, presence of chronic conditions such as diabetes and hypertension]; and reproductive health indicators (menstrual cycle regularity).

Specific categorization of these covariates was as follows:

- Race/ethnicity was categorized into five groups: Mexican American, Other Hispanic, non-Hispanic White, non-Hispanic Black, and Other Race.
- Education level was classified into three categories: less than high school, high school graduate, and more than high school.
- Marital status was grouped into: married/living with a partner, divorced/separated/widowed, and never married.
- Body Mass Index (BMI) was categorized as: normal weight (BMI < 25), overweight (25 ≤ BMI ≤ 30), and obese (BMI > 30).
- Poverty Income Ratio (PIR): reflecting socioeconomic status by comparing household income to the federal poverty threshold, was divided into three levels: low (PIR < 1.35), medium (1.35 ≤ PIR ≤ 3.0), and high (PIR > 3.0) [18].
- Smoking status was classified according to the National Health and Nutrition Examination Survey (NHANES) detailed records on participants’ smoking behavior, including status, duration, and related activities. The categories were defined as: never smokers (individuals who have never smoked or have smoked fewer than 100 cigarettes in their lifetime); former smokers (individuals who have smoked 100 or more cigarettes in their lifetime but are currently not smoking); and current smokers (individuals who have smoked 100 or more cigarettes in their lifetime and are still smoking, regardless of frequency) [30].
- Diabetes was assessed through a questionnaire asking participants, “Has a doctor or other health professional ever told you that you have diabetes?” Those who answered affirmatively were classified as having diabetes [31].
- Hypertension was determined by a similar questionnaire question: “Has a doctor or other health professional ever told you that you have high blood pressure?” Participants who answered “yes” were classified as having hypertension.
- Menstrual cycle regularity was judged based on a question in the reproductive health questionnaire: “Has your menstrual cycle been regular in the past 12 months?” (applicable to participants aged 12 years and older).

Missing covariate data were handled using multiple imputation. The imputation model included variables used in the main analysis, and each imputed dataset was subsequently analyzed under the NHANES complex survey design; the estimates were then combined according to Rubin’s rules. All analyses were performed in R 4.4.3.

### 2.5. Machine Learning

To further explore the relative association of each component of the CDAI with the prevalence of PID under the control of confounding factors, this study employed the Random Forest algorithm for machine learning modeling. Importantly, the machine-learning analysis was exploratory and was used to characterize model-based variable importance rather than to identify mechanisms or develop a deployable prediction model. This design provides a robust methodological basis for uncovering complex, non-linear nutritional interaction networks. Random Forest is an ensemble learning method that builds multiple decision tree models and aggregates their results to enhance predictive accuracy. Each tree is constructed using Bootstrap sampling to randomly select a training subset, and at each node, a random subset of features is selected for splitting, thereby enhancing the model’s generalization capability. Its advantages include effectively reducing the risk of overfitting and demonstrating strong robustness to missing values and high-dimensional data [32].

To ensure robust model evaluation and avoid data leakage, the complete dataset was randomly partitioned into a training set (70%) for model derivation and an independent testing set (30%) for validation. In this study, we incorporated dietary antioxidant components (vitamin A, C, E, carotenoids, zinc, and selenium) along with all covariates into the model and constructed a random forest classifier consisting of 500 trees using the training cohort. Subsequently, we utilized SHAP (SHapley Additive exPlanations) values, which were extracted exclusively from the independent testing set, to evaluate the relative contribution of each variable to the prediction of PID.

### 2.6. Statistical Analysis

All analyses were conducted in accordance with the NHANES analytic guidelines for complex multistage sampling. Participants were categorized according to whether they reported a history of treated PID. Covariate adjustment was performed using a sequential modeling strategy. Model 1 was an unadjusted survey-weighted logistic regression model. Model 2 adjusted for demographic, socioeconomic, adiposity-related, reproductive, and chronic disease variables, including age, race/ethnicity, education level, marital status, poverty income ratio (PIR), body mass index (BMI), menstrual regularity, hypertension, and diabetes. Model 3 was the fully adjusted model and further included smoking status because smoking was considered an important potential confounder. Age was entered as a continuous variable, whereas categorical covariates were entered as indicator variables according to the predefined categories described above. For the quartile-based analyses, the lowest CDAI quartile was used as the reference group. The same fully adjusted covariate set was applied in the restricted cubic spline analysis, CDAI component analyses, and subgroup analyses unless otherwise specified. To evaluate potential selection bias arising from sample exclusion, we additionally compared baseline characteristics between the final analytic sample and RHQ078-eligible women who were excluded because of missing, refused, or “don’t know” responses to RHQ078 or incomplete information required for CDAI calculation or smoking status. Continuous variables were compared using Welch tests, and categorical variables were compared using chi-square tests. The results are presented in Supplementary Table S1. Baseline characteristics of the population were described using mean ± standard deviation for continuous variables and frequency and percentage for categorical variables. To calculate the *p*-value for trend, CDAI was divided into quartiles, and the median of each quartile was treated as a continuous variable in logistic regression models. Restricted cubic spline models were fitted to evaluate the exposure–response association between CDAI and self-reported history of treated PID. This method is commonly used in dose–response analyses of continuous exposures because it preserves the continuous scale of the exposure and permits flexible modeling of potential nonlinear associations. Restricted cubic spline models were fitted to evaluate the exposure–response association between CDAI and self-reported history of treated PID. Three knots were placed at the 10th, 50th, and 90th percentiles of the CDAI distribution in the analytic sample, and the median CDAI value was used as the reference. To reduce instability driven by sparse extreme values, CDAI values outside the 1st and 99th percentiles were truncated before spline fitting. Overall and nonlinearity *p*-values were derived from Wald tests. Subgroup analyses were further conducted to explore this relationship, stratified by variables including age, BMI, smoking status, education level, PIR, race/ethnicity, marital status, menstrual regularity, hypertension, and diabetes.

All statistical analyses were performed in R software (version 4.4.3). The NHANES complex sampling design was accounted for by incorporating sample weights, masked strata, and primary sampling units, and variance estimates were obtained using Taylor series linearization. A two-sided *p*-value < 0.05 was considered statistically significant. For the combined analysis of NHANES 2013–2018, the corresponding 2-year dietary sample weights were divided by 3 to generate 6-year combined weights, following NHANES analytic recommendations.

## 3. Results

### 3.1. Participant Characteristics

A total of 4539 female participants were included in the final analysis, with a mean age of 38.31 years. Among them, 5.6% reported a history of pelvic inflammatory disease. Among the RHQ078-eligible target population, 895 women were excluded from the final analytic sample. Compared with excluded RHQ078-eligible women, included participants were slightly older (38.31 vs. 36.96 years, *p* = 0.002) and differed significantly in race/ethnicity, education level, poverty income ratio, BMI, smoking status, menstrual regularity, and CDAI availability. As expected, CDAI availability differed markedly because missing CDAI was a principal exclusion criterion (100.0% vs. 3.6%, *p* < 0.001); among participants with available CDAI values, mean CDAI did not differ significantly between the included and excluded groups (0.64 vs. 1.13, *p* = 0.528). In contrast, marital status, diabetes, and hypertension did not differ significantly between groups. The PID response distribution differed because the excluded group contained refused or “don’t know” responses to RHQ078. These comparisons are shown in Supplementary Table S1.

Table 1 presents the baseline characteristics of participants according to PID status. Significant differences (*p* < 0.05) were observed between the two groups across multiple variables, including age, smoking status, marital status, race/ethnicity, PIR, BMI, menstrual regularity, history of hypertension, and CDAI levels.

### 3.2. The Association Between CDAI and PID

In quartile-based weighted logistic regression, the inverse association between CDAI and self-reported history of treated PID was attenuated after progressive adjustment (Table 2). In Model 2, compared with Q1, Q2 and Q3 were associated with lower odds of PID, whereas Q4 did not reach statistical significance (OR = 0.666, 95% CI: 0.380–1.167, *p* = 0.167), although the trend test was significant (*p* for trend = 0.029). After further adjustment for smoking status in Model 3, only Q3 remained statistically significant (OR = 0.576, 95% CI: 0.374–0.886, *p* = 0.019), while Q2, Q4, and the trend test were no longer statistically significant. These results suggest an inverse but non-monotonic and smoking-sensitive association, and they do not support a statistically significant reduction in PID odds among participants in the highest CDAI quartile in the fully adjusted quartile model.

Furthermore, we observed that after adjusting for all selected confounders except for smoking status, followed by the adjustment for smoking status (Model 3), the *p*-value for the dose–response trend significantly increased. This finding suggests that the observed association between CDAI and self-reported history of treated PID was attenuated after adjustment for smoking status, indicating potential confounding by smoking. Moreover, the third quartile (Q3) of CDAI may represent a critical threshold for further exploration of its biological mechanisms in relation to PID. The confounding effect of smoking within this association warrants separate, in-depth analysis.

**Figure 2:**
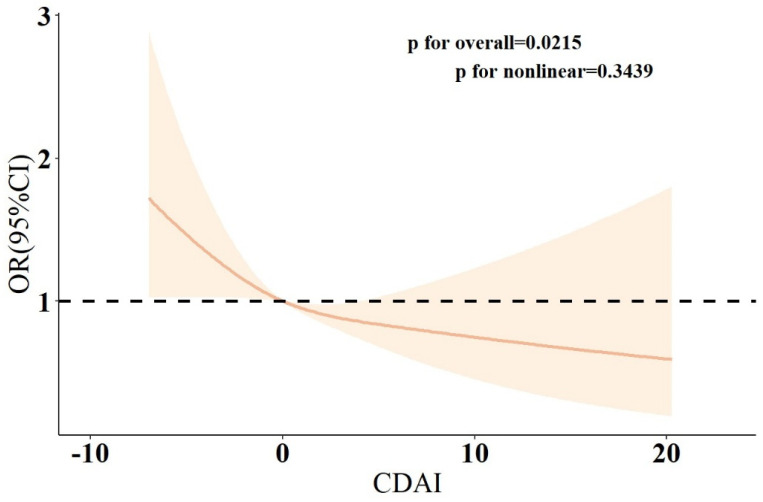
RCS fitted curve illustrating the association between CDAI and PID.

RCS analysis indicated an overall inverse association between CDAI and the odds of self-reported history of treated PID (overall *p* = 0.0215), with no evidence of significant nonlinearity (*p* for nonlinearity = 0.3439) (Figure 2). These findings are compatible with an approximately linear inverse association between CDAI and self-reported history of treated PID, although nonlinearity cannot be definitively excluded.

Model 1: Unadjusted model. Model 2: Adjusted for age, race/ethnicity, education level, marital status, poverty-income ratio (PIR), body mass index (BMI), regular menstrual cycle, hypertension, and diabetes. Model 3: Adjusted for age, race/ethnicity, education level, marital status, poverty-income ratio (PIR), body mass index (BMI), regular menstrual cycle, smoking status, hypertension, and diabetes. Abbreviations: CI, confidence interval; OR, odds ratio; Ref, reference. CDAI Quartiles: Q1 (<−1.899), Q2 (−1.899 to 0.284), Q3 (0.284 to 2.751), Q4 (>2.751).

RCS modeling was used to flexibly evaluate CDAI as a continuous exposure, avoiding information loss caused by arbitrary categorization and allowing assessment of both the overall association and potential nonlinearity. The solid line represents the estimated odds ratio (OR), and the shaded area represents the 95% confidence interval (CI). The horizontal dashed line indicates OR = 1. The overall *p*-value evaluates whether CDAI is associated with PID across the exposure distribution, whereas the *p*-value for nonlinearity evaluates whether the association departs from a linear pattern. Models were adjusted for age, race/ethnicity, education level, marital status, poverty-income ratio (PIR), body mass index (BMI), menstrual regularity, smoking status, hypertension, and diabetes.

### 3.3. Association Between CDAI Components and PID

We further analyzed the independent relationships between the six antioxidant components of CDAI and the risk of PID.

As shown in Table 3, in the model adjusted for all covariates (including smoking status):

■ Vitamin E showed a significant inverse association with PID (OR = 0.963, 95% CI: 0.933–0.995, *p* = 0.032).
■ The remaining components, including vitamin A (*p* = 0.151), vitamin C (*p* = 0.312), zinc (*p* = 0.310), selenium (*p* = 0.220), and carotenoids (*p* = 0.405), did not demonstrate statistically significant associations with PID risk.

These results suggest that although CDAI as a composite index was associated with the prevalence of self-reported history of treated PID, the associations of its individual components were heterogeneous, with vitamin E showing the most consistent inverse association in the fully adjusted regression model.

RCS analysis suggested an L-shaped nonlinear association between vitamin A intake and the odds of self-reported history of treated PID, whereas selenium intake showed a U-shaped association. These nonlinear patterns should be interpreted cautiously because estimates at the tails of the exposure distribution may be less stable. In the fully adjusted component-specific regression models, only vitamin E showed a statistically significant inverse association with self-reported history of treated PID.

Model 1: Unadjusted model. Model 2: Adjusted for age, race/ethnicity, education level, marital status, poverty-income ratio (PIR), body mass index (BMI), regular menstrual cycle, hypertension, and diabetes. Model 3: Adjusted for age, race/ethnicity, education level, marital status, poverty-income ratio (PIR), body mass index (BMI), regular menstrual cycle, smoking status, hypertension, and diabetes.

**Figure 3:**
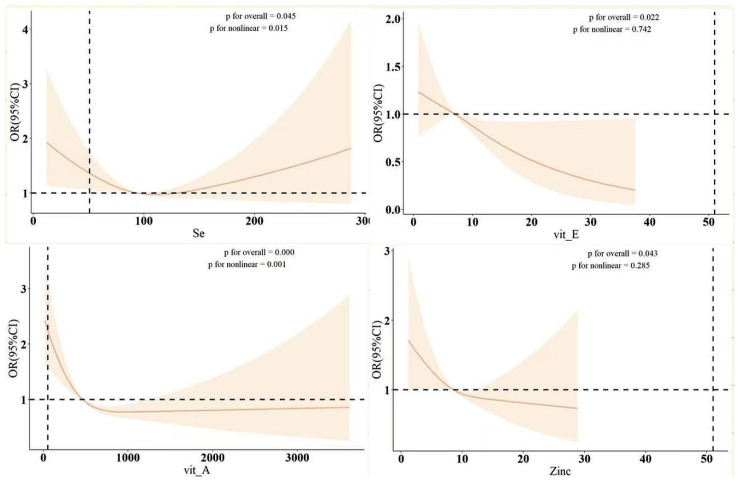
Restricted cubic spline analysis of the associations between individual CDAI components and self-reported history of treated PID.

Figure 3: RCS fitted curve illustrating the association between CDAI components and PID.

The solid lines represent fitted odds ratios, and the shaded areas represent 95% confidence intervals. Models were adjusted for age, race/ethnicity, education level, marital status, poverty income ratio, body mass index, menstrual regularity, smoking status, hypertension, and diabetes. These component-specific RCS analyses were exploratory and should be interpreted cautiously.

### 3.4. SHAP-Based Ranking of Antioxidant Contributions from CDAI Components

**Figure 4:**
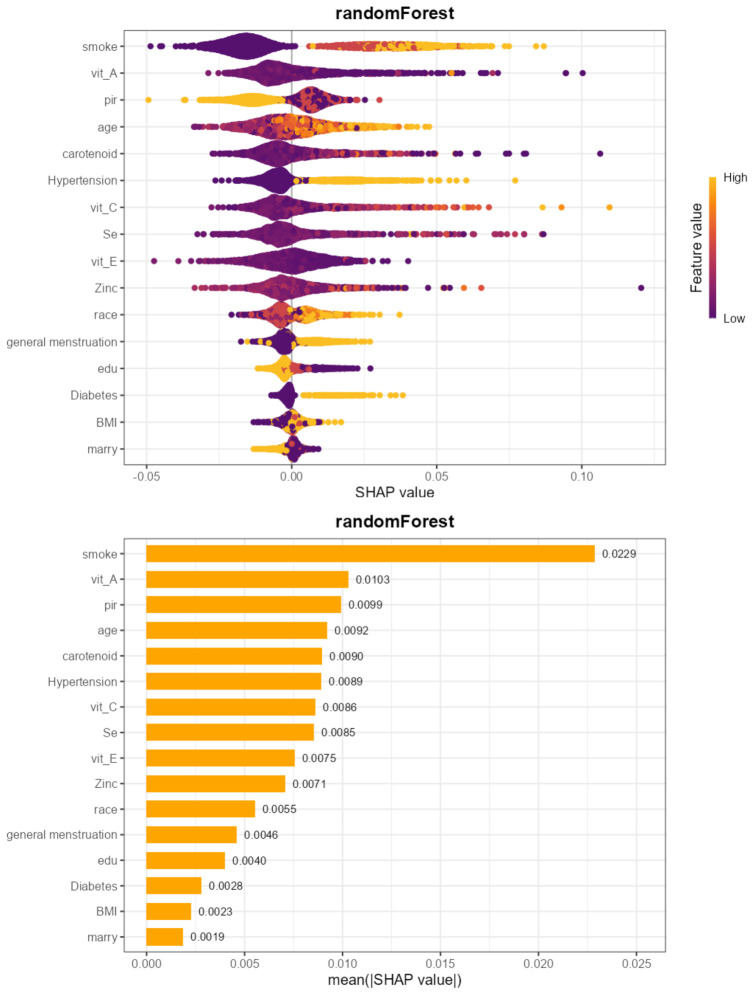
SHAP Value Ranking in the Random Forest Model (bar plot and beeswarm plot).

By integrating the random forest model with SHAP (SHapley Additive exPlanations) values analysis, we further evaluated the model-based importance ranking of CDAI components for classifying self-reported history of treated PID (see Figure 4).

The results showed that:

● Vitamin A, carotenoids, and vitamin C exhibited the highest SHAP values among CDAI components, ranking first, second, and third, respectively. This ranking indicates that these components had the largest model-based contributions among CDAI components to PID classification in the random forest model, highlighting nutrients that may warrant further investigation in future mechanistic or longitudinal studies.
● Furthermore, smoking status had the largest mean absolute SHAP value, suggesting the greatest model-based contribution to PID classification among the included variables.
● This suggests that, although vitamin E showed a significant inverse association with PID in the component-specific fully adjusted regression model, the random forest model assigned higher predictive contributions to vitamin A, carotenoids, and vitamin C among CDAI components. This discrepancy may reflect differences between regression-based marginal association estimates and model-based predictive attribution, and should be interpreted as exploratory rather than causal evidence.

Figure 4: SHAP value ranking in the random forest model. The SHAP summary plot and bar plot show the relative contribution of each variable to the model prediction of self-reported history of treated PID. Variables with higher mean absolute SHAP values contributed more strongly to model prediction, suggesting greater relative importance within the multivariable random forest framework. SHAP rankings should be interpreted as model-based predictive contributions rather than causal effects.

### 3.5. Subgroup Analysis

**Figure 5:**
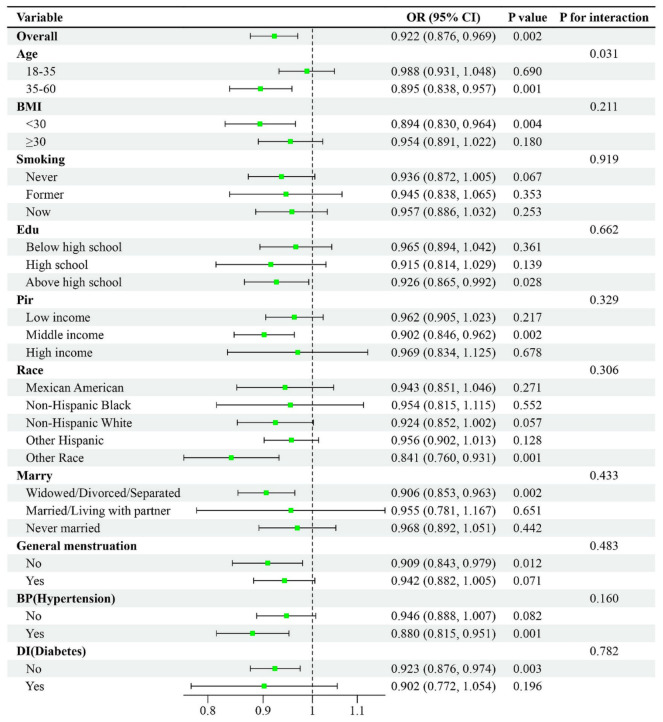
Subgroup analysis for the association between CDAI and PID.

To explore potential heterogeneity in the association between CDAI and self-reported history of treated PID, subgroup analyses were conducted (see Figure 5).

- Significant inverse associations between CDAI and PID risk were observed in the following subgroups: age 35–60 years, BMI < 30, education level above high school, middle-income, Other Race, divorced/widowed/separated, absence of menstruation, and hypertension.
- The interaction *p*-value for age in the model was 0.031, indicating a significant difference in the association of CDAI with PID across age groups.
- Interaction terms for other variables, such as BMI, smoking, income, marital status, and chronic disease status, all had *p*-values > 0.05, suggesting that the overall trend remained consistent across most subgroups.

## 4. Discussion

In this nationally representative cross-sectional analysis of U.S. women, we observed a suggestive inverse association between CDAI and self-reported history of treated PID. However, the evidence was not uniform across analytic approaches. The restricted cubic spline model suggested an overall inverse association without strong evidence of nonlinearity, whereas the quartile-based fully adjusted logistic regression showed that the highest CDAI quartile was not statistically significant and that the trend was attenuated after adjustment for smoking. Therefore, the findings should be interpreted as exploratory observational evidence rather than as confirmation of a protective effect of high dietary antioxidant intake against PID. Our findings should be interpreted within the broader international literature on reproductive tract inflammation, sexually transmitted infections, bacterial vaginosis, and vaginal microbiota. Recent studies suggest that BV and vaginal dysbiosis are associated with PID and other upper genital tract inflammatory conditions, while dietary patterns, micronutrient intake, and dietary antioxidant capacity may be related to BV risk or severity. These data provide indirect but biologically relevant context for the observed association between CDAI and PID history [33,34,35].

Human epidemiological evidence directly linking diet with PID remains limited, but related literature on reproductive tract inflammation and vaginal dysbiosis provides useful context. In NHANES, a higher Dietary Inflammatory Index has been associated with greater odds of PID, supporting the relevance of diet-related inflammatory potential to PID epidemiology [36]. In addition, bacterial vaginosis, a lower-genital-tract dysbiosis associated with subsequent PID, has been linked to dietary exposures in observational studies [34]. Prior studies reported that higher dietary fat intake or glycemic load and poorer nutrient density were associated with BV or BV persistence [37], whereas healthier or plant-based dietary patterns, higher micronutrient intake, Mediterranean diet adherence, and dietary total antioxidant capacity were associated with lower BV odds or severity [35]. These human data provide an epidemiological framework for our findings and suggest that antioxidant-rich dietary patterns may be related to reproductive tract inflammatory susceptibility. Nevertheless, these studies, including ours, are observational; therefore, they cannot determine whether dietary modification reduces incident PID.

These findings support an inverse association between antioxidant-rich dietary patterns and the prevalence of self-reported history of treated PID, and they provide observational evidence to inform future longitudinal and interventional studies. Additionally, subgroup analysis results indicated a significant interaction effect of age in the association between antioxidant dietary patterns and PID risk.

Previous studies have extensively explored PID prevention and treatment. For instance, preclinical rat-model evidence suggests potential anti-inflammatory and antioxidant effects of acetyl-L-carnitine in PID-related inflammation [38]. Furthermore, other studies have identified the beneficial effects of magnesium supplementation in reducing inflammatory biomarkers [39]. These prior discoveries underscore the potential value of dietary factors in mitigating PID risk, lending biological plausibility to our investigation of antioxidant-rich diets and their inverse association with PID prevalence.

PID is a common condition affecting women worldwide. It is a chronic inflammatory disorder caused by the ascending spread of pathogens from the vagina to the uterus, which can lead to severe consequences such as infertility [33]. A review of PID diagnosis [40] not only highlighted the lack of specificity in clinical diagnosis but also revealed that less than half of the women diagnosed with PID actually had the condition. Furthermore, Mycoplasma genitalium, respiratory pathogens, and a group of bacteria associated with bacterial vaginosis account for a substantial proportion of PID cases. This underscores the urgent need for more accurate diagnostic techniques for PID and indirectly emphasizes the importance of preventive strategies. This study found a significant inverse association between CDAI and the prevalence of self-reported history of treated PID, suggesting that antioxidant-rich dietary patterns may be correlated with lower PID prevalence at the population level.

Dietary prevention has gained attention over time, and several studies have explored the role of diet in the treatment and prevention of PID. Chen’s study [18] found a significant inverse association between magnesium intake and PID prevalence, Hui’s research [17] indicated a certain link between mineral intake and PID risk, and Wang [41] demonstrated that engelitin, a compound found in plant-based foods such as fruits and vegetables, exhibits anti-PID activity. Although these studies provide new theoretical support for dietary interventions, they did not examine the linear relationship between dietary factors and PID prevalence. In contrast to previous cross-sectional studies on diet and PID, we incorporated a random forest machine learning model and used restricted cubic splines (RCSs) to demonstrate a linear association between CDAI and PID prevalence (*p* for nonlinear = 0.3439 > 0.05).

However, it is undeniable that machine learning models often suffer from low interpretability [42]. To address this, we used SHAP to enhance the transparency and interpretability of the model [43]. The SHAP summary plot revealed that smoking had the largest model-based contribution to PID prediction, followed by vitamin A, PIR, age, carotenoids, hypertension, vitamin C, and selenium.

This apparent discrepancy between the logistic regression and SHAP results stems from fundamental differences in how generalized linear models and tree-based ensemble models represent data structure and feature effects [44,45,46]. Logistic regression is a generalized linear parametric model that estimates the independent marginal effect of a predictor on the log-odds scale, and its interpretability depends heavily on reasonable handling of multicollinearity, functional form, and interaction terms [44]. In contrast, tree-based ensemble methods such as Random Forest do not assume linearity and can accommodate complex threshold effects and higher-order interactions through recursive partitioning and aggregation [45,46]. Accordingly, SHAP values—grounded in cooperative game theory—estimate feature contributions across many feature coalitions rather than in isolation, making them especially sensitive to nonlinear dependence structures and interaction effects that conventional linear specifications may underrepresent or miss [46].

Therefore, this divergence is better interpreted not as a statistical artifact but as a reflection of the biological complexity of antioxidant exposure and redox regulation. The independent significance of vitamin E in the logistic regression is biologically plausible because vitamin E is a major lipid-phase chain-breaking antioxidant with a well-established direct antioxidant role [22]. By contrast, the prominence of vitamin C and selenium in the SHAP analysis is consistent with an interdependent antioxidant network rather than isolated single-nutrient effects [23,24,25]. Physiologically, vitamin C can reduce the tocopheroxyl radical and thereby regenerate vitamin E, whereas selenium is incorporated into glutathione peroxidases, whose catalytic cycling depends on glutathione-derived reducing equivalents to maintain antioxidant defense [23,47,48]. Traditional regression models can attenuate or mask such correlated and nonlinear joint effects when these nutrients are decomposed into separate main-effect terms, whereas machine learning models are better positioned to recover these interaction-rich patterns [45,46]. Because the mechanistic evidence for vitamin A as a direct member of the classic vitamin C–vitamin E–selenium antioxidant circuit is less definitive, it is safer to interpret its SHAP prominence as a marker of broader dietary redox/nutrient patterning rather than as proof of the same canonical synergistic pathway [49].

Consistent with our findings, existing studies have confirmed that smoking is associated with the diagnosis of bacterial vaginosis and a vaginal microbiota lacking protective Lactobacillus species [50]. The disruption of Lactobacillus dominance increases the risk of sexually transmitted infections and upper genital tract infections [51], thereby contributing to the development of PID. This may partly explain the significant impact of smoking.

This study, using a nationally representative sample from NHANES, identified an inverse association between CDAI and self-reported history of treated PID. Higher CDAI scores were associated with a lower prevalence of PID, and subgroup analyses suggested generally similar directions across several demographic and clinical strata. These findings indicate that antioxidant-rich dietary patterns may be relevant to reproductive health promotion and warrant further evaluation in prospective and interventional studies.

Supported by machine learning analysis, our findings further identified the model-based importance ranking of CDAI components, with vitamin A, carotenoids, and vitamin C showing the highest predictive contributions among the antioxidant components. These findings may reflect complex dietary patterns and potential nonlinear relationships, but they should not be interpreted as evidence of biological synergy. Additionally, the significant impact of behavioral factors, notably smoking, on PID risk underscores the necessity of integrating nutritional interventions with the management of healthy behavioral practices.

Based on these findings, we recommend that the public increase their dietary intake of naturally antioxidant-rich foods, including leafy green vegetables, berries, fruits, nuts, and whole grains, while reducing risk behaviors such as smoking, as part of dietary prevention of PID and overall health improvement.

Future research should aim to validate and quantify the long-term protective effects of antioxidant diets on PID through longitudinal studies and interventional trials. Mechanistic exploration should be deepened by integrating data on the microbiome, biochemical markers, and individual nutrient metabolism, thereby providing a scientific foundation for the development of precision nutrition strategies.

## 5. Study Limitations

Several limitations of this study should be highlighted.

First, because of the cross-sectional design and the temporal discordance between exposure and outcome assessment, causality and temporality cannot be established. CDAI was calculated from two 24 h dietary recalls collected during the NHANES examination period, whereas RHQ078 ascertained whether participants had ever been treated for an infection in the fallopian tubes, uterus, or ovaries, also called PID. Therefore, CDAI reflects current reported dietary antioxidant intake rather than dietary exposure before PID onset. Participants with a previous PID diagnosis may have changed their diet, supplement use, smoking behavior, health-care use, or other lifestyle factors after medical advice or symptom experience. Reverse causation is therefore a central limitation of the present analysis, and the observed association should be interpreted as a cross-sectional correlation with self-reported history of treated PID rather than evidence that higher CDAI prevents PID. This association needs to be validated through larger prospective cohort studies.

Second, residual confounding by sexual behavior and STI history cannot be excluded. Active or previous infections with Chlamydia trachomatis and Neisseria gonorrhoeae, bacterial vaginosis, condom use, number of sexual partners, and other sexual behaviors are established or biologically plausible determinants of PID. Although NHANES contains several sexual behavior and self-reported STI variables, these variables were not consistently available in the public-use files across all cycles included in the present 2013–2018 analysis, because the 2017–2018 adult sexual behavior file is restricted-access. Consequently, we could not include these variables in the primary public-use analysis without either restricting the study period or using RDC data. This limitation may have resulted in residual confounding and should be considered when interpreting the observed association between CDAI and PID.

Thirdly, although recent international literature was incorporated to contextualize the findings, the analytic sample was restricted to U.S. women from NHANES. Therefore, the observed association may not be directly generalizable to populations in regions with different dietary structures, micronutrient intake profiles, STI epidemiology, vaginal microbiota patterns, healthcare access, and PID diagnostic practices. Future studies from diverse geographic settings are needed to validate these findings [6,33,34].

Fourth, PID was defined using self-reported treatment history rather than clinical verification, which may have introduced outcome misclassification, including underascertainment of asymptomatic cases and misclassification of other pelvic conditions. Additionally, self-reporting bias cannot be entirely eliminated.

Fifth, dietary intake was assessed using 24 h dietary recalls, which may not fully reflect long-term habitual intake and may be subject to recall error; therefore, some degree of exposure misclassification is unavoidable.

Lastly, SHAP values reflect the local influence of a specific feature on model predictions and may not fully capture global non-linear relationships or higher-order interaction effects. As a result, the synergistic effects among CDAI components might not be fully elucidated.

## 6. Conclusions

In this nationally representative cross-sectional study of U.S. women, higher CDAI was inversely associated with the prevalence of self-reported history of treated PID. The association appeared generally linear in restricted cubic spline analysis and was more evident in several subgroups, particularly middle-aged and older women. Among individual CDAI components, vitamin E showed an independent inverse association in weighted logistic regression, whereas SHAP-based random forest interpretation suggested that vitamin A, carotenoids, and vitamin C had the highest predictive contributions among CDAI components. These findings support a potential link between antioxidant-rich dietary patterns and lower PID prevalence, but causal inference cannot be made because of the cross-sectional design and self-reported outcome definition. Future prospective studies and mechanistic investigations are needed to validate these findings and clarify the biological pathways underlying the observed association.

## Figures and Tables

**Table 1:** Characteristics of the study population based on the presence of PID.

Variable	Overall (*n* = 4539)	Non-PID (*n* = 4284)	PID (*n* = 255)	*p*
**age**	38.31 (12.38)	38.06 (12.44)	42.46 (11.07)	<0.001
**smoke (%)**				<0.001
Never	3163 (69.7)	3052 (71.2)	111 (43.5)	
Former	597 (13.2)	541 (12.6)	56 (22.0)	
Now	779 (17.2)	691 (16.1)	88 (34.5)	
**marry (%)**				<0.001
Widowed/Divorced/Separated	2657 (58.5)	2519 (58.8)	138 (54.1)	
Married/Living with partner	817 (18.0)	741 (17.3)	76 (29.8)	
Never married	1065 (23.5)	1024 (23.9)	41 (16.1)	
**race (%)**				<0.001
Mexican American	733 (16.2)	711 (16.6)	22 (8.6)	
Non-Hispanic Black	490 (10.8)	466 (10.9)	24 (9.4)	
Non-Hispanic White	1565 (34.5)	1468 (34.3)	97 (38.0)	
Other Hispanic	1043 (23.0)	962 (22.5)	81 (31.8)	
Other Race	708 (15.6)	677 (15.8)	31 (12.2)	
**edu (%)**				0.314
Below high school	713 (15.7)	667 (15.6)	46 (18.0)	
High school	921 (20.3)	864 (20.2)	57 (22.4)	
Above high school	2905 (64.0)	2753 (64.3)	152 (59.6)	
**pir (%)**				<0.001
low income	1605 (35.4)	1493 (34.9)	112 (43.9)	
middle income	1636 (36.0)	1536 (35.9)	100 (39.2)	
high income	1298 (28.6)	1255 (29.3)	43 (16.9)	
**BMI (%)**				0.001
normal	1457 (32.1)	1399 (32.7)	58 (22.7)	
overweight	1118 (24.6)	1056 (24.6)	62 (24.3)	
obesity	1964 (43.3)	1829 (42.7)	135 (52.9)	
**Hypertension = Yes (%)**	1044 (23.0)	944 (22.0)	100 (39.2)	<0.001
**Diabetes = Yes (%)**	351 (7.7)	325 (7.6)	26 (10.2)	0.163
**general menstruation = Yes (%)**	3166 (69.8)	3022 (70.5)	144 (56.5)	<0.001
**CDAI**	0.63 (4.13)	0.68 (4.14)	−0.20 (3.89)	0.001
**vit_A**	559.40 (457.20)	563.24 (455.44)	494.32 (490.27)	0.019
**vit_C**	74.27 (65.01)	74.59 (64.94)	68.59 (66.25)	0.153
**vit_E**	8.08 (5.18)	8.13 (5.21)	7.28 (4.75)	0.011
**Zinc**	9.21 (4.20)	9.25 (4.22)	8.47 (3.93)	0.004
**Se**	99.55 (43.95)	99.96 (44.11)	93.00 (41.19)	0.014
**carotenoid**	8602.20 (9314.00)	8654.00 (9400.81)	7339.28 (7677.76)	0.029

Note: Continuous variables are presented as mean (standard deviation), and categorical variables are presented as *n* (%). The values in parentheses indicate standard deviations for continuous variables and percentages for categorical variables. Percentages were calculated within each corresponding PID-status column.

**Table 2:** The weighted logistic regression analysis of the association between CDAI and PID.

	Model 1		Model 2		Model 3	
CDAI	OR (95% CI)	*p*-value	OR (95% CI)	*p*-value	OR (95% CI)	*p*-value
Q1	Ref		Ref		Ref	
Q2	0.593 (0.432, 0.813)	0.006	0.682 (0.485, 0.959)	0.036	0.731 (0.523, 1.023)	0.079
Q3	0.417 (0.286, 0.609)	<0.001	0.524 (0.334, 0.819)	0.009	0.576 (0.374, 0.886)	0.019
Q4	0.473 (0.282, 0.827)	0.014	0.666 (0.380, 1.167)	0.167	0.744 (0.429, 1.292)	0.304
*p* for trend	<0.001		0.029		0.076	

**Table 3:** The weighted logistic regression analysis of the association between CDAI components and PID.

	Model 1		Model 2		Model 3	
	OR (95% CI)	*p*-value	OR (95% CI)	*p*-value	OR (95% CI)	*p*-value
VIT-A	0.999 (0.998, 1.000)	0.048	0.999 (0.998, 1.000)	0.144	0.999 (0.998, 1.000)	0.151
VIT-C	1.000 (0.997, 1.003)	0.863	1.002 (0.998, 1.006)	0.302	1.002 (0.998, 1.006)	0.312
VIT-E	0.952 (0.915, 0.990)	0.018	0.964 (0.933, 0.996)	0.032	0.963 (0.933, 0.995)	0.032
Zinc	0.981 (0.922, 1.043)	0.541	0.971 (0.916, 1.030)	0.329	0.970 (0.915, 1.027)	0.310
Se	1.002 (0.996, 1.008)	0.461	1.003 (0.998, 1.009)	0.238	1.004 (0.998, 1.009)	0.220
Carotenoid	1.000 (0.999, 1.000)	0.433	1.000 (0.999, 1.000)	0.394	1.000 (0.999, 1.000)	0.405

## Data Availability

The data used in this study are publicly available from the National Health and Nutrition Examination Survey (NHANES) database.

## Supplementary Materials

The following supporting information can be downloaded at: https://www.mdpi.com/article/10.3390/healthcare14121682/s1, Table S1: Baseline characteristics of included participants and excluded RHQ078-eligible women in NHANES 2013–2018.
